# Elastic Energy Storage in Al–Al_4_C_3_ Composites: Effects of Dislocation Character and Interfacial Graphite Formation

**DOI:** 10.3390/ma19010181

**Published:** 2026-01-04

**Authors:** Audel Santos Beltrán, Verónica Gallegos Orozco, Hansel Manuel Medrano Prieto, Ivanovich Estrada Guel, Carlos Gamaliel Garay Reyes, Miriam Santos Beltrán, Diana Verónica Santos Gallegos, Carmen Gallegos Orozco, Roberto Martínez Sánchez

**Affiliations:** 1 Departamento de Nanotecnología, Universidad Tecnológica de Chihuahua Sur, Km. 3.5 Carr. Chihuahua a Aldama, Chihuahua 31313, Mexico; hmedrano@utchsur.edu.mx (H.M.M.P.); msantos@utchsur.edu.mx (M.S.B.);; 2 División de Estudios de Posgrado e Investigación, Tecnológico Nacional de México Campus Chihuahua, Av. Tecnológico 2909, Chihuahua 31200, Mexico; 3 Centro de Investigación en Materiales Avanzados (CIMAV), Laboratorio Nacional de Nanotecnología, Miguel de Cervantes No. 120, Chihuahua 31136, Mexico; ivanovich.estrada@cimav.edu.mx (I.E.G.); carlos.garay@cimav.edu.mx (C.G.G.R.); roberto.martinez@cimav.edu.mx (R.M.S.); 4 Departamento de Económico Administrativo, Tecnológico Nacional de México, Campus Chihuahua II, Ave. de las Industrias #11101, Complejo Industrial Chihuahua, Chihuahua 31130, Mexico

**Keywords:** Al–Al_4_C_3_ composites, elastic energy storage, dislocation character (screw/edge)

## Abstract

Al–Al_4_C_3_ composites exhibit promising mechanical properties including high specific strength, high specific stiffness. However, high reinforcement contents often promote brittle behavior, making it necessary to understand the mechanisms governing their limited toughness. In this work, a microstructural and mechanical study was carried out to evaluate the energy storage capacity in Al–Al_4_C_3_ composites fabricated by mechanical milling followed by heat treatment using X-ray diffraction (XRD) and Convolutional Multiple Whole Profile (CMWP) fitting method, the microstructural parameters governing the initial stored energy after fabrication were determined: dislocation density (*ρ*), dislocation character (*q*), and effective outer cut-off radius (R_e_). Compression tests were carried out to quantify the elastic energy stored during loading (Es). The energy absorption efficiency (*EAE*) in the elastic region of the stress–strain curve was evaluated with respect to the elastic energy density per unit volume stored (*Ee*), obtained from microstructural parameters (*ρ*, *q*, and *Re*) present in the samples after fabrication and determined by XRD. A predictive model is proposed that expresses *Es* as a function of *Ee* and *q*, where the parameter *q* is critical for achieving quantitative agreement between both energy states. In general, samples with high *EAE* exhibited microstructures dominated by screw-character dislocations. High-resolution transmission electron microscopy (HRTEM) analyses revealed graphite regions near Al_4_C_3_ nanorods—formed during prolonged sintering—which, together with the thermal mismatch between Al and graphite during cooling, promote the formation of screw dislocations, their dissociation into extended partials, and the development of stacking faults. These mechanisms enhance the redistribution of stored energy and contribute to improved toughness of the composite.

## 1. Introduction

Aluminum-based metal matrix composites (MMCs) have attracted significant attention in the aerospace, automotive, and defense industries due to their excellent combination of properties, including high specific strength, high specific stiffness, good thermal conductivity, wear resistance, and outstanding corrosion resistance compared to monolithic materials. These characteristics, together with their high strength-to-density ratio, good ductility, and manufacturability, make them ideal candidates for critical structural applications [1,2,3,4]. Among fabrication techniques, high-energy mechanical milling stands out as an effective method for producing MMCs through powder metallurgy. This solid-state process promotes a homogeneous distribution of reinforcements through repeated welding and fracturing cycles of particles, resulting in nanocrystalline microstructures that enhance mechanical strength [5,6,7,8]. Optimization of milling parameters, such as rotational speed, makes it possible to tune strength and ductility, obtaining ultrahigh-strength or ultraductile nanocomposites from the same composition [9,10,11,12,13]. Given these materials’ exposure to dynamic loads—such as impacts in aerospace structures or armor—it is essential to evaluate properties like fracture toughness and energy-absorption capacity [14]. Recent studies have shown that nanoreinforcements not only improve strength and stiffness but also simultaneously increase energy absorption and the ability to dissipate vibrations—critical aspects for preventing brittle failures in service [14]. The reinforcement type and content are determining factors. For example, a 40% volume fraction of SiCp/2024Al exhibits ductile behavior, whereas a 50% volume fraction yields predominantly brittle behavior, evidencing a critical content for the transition between these regimes [15]. Likewise, the incorporation of nanometric reinforcements such as carbon nanotubes (CNTs), graphene, or fullerenes improves tribological behavior and wear resistance, but excessive additions can cause agglomeration and loss of mechanical properties [16]. The fabrication technique also directly affects performance. In carbon-fiber–reinforced aluminum (CF/Al) composites, indirect extrusion has shown significant increases in compressive strength, fracture toughness, and fracture work compared with direct extrusion, due to better reinforcement distribution and intermediate alloy layers that facilitate energy absorption [17]. In hybrid systems—such as Al–Mg composites reinforced with ammonium polyphosphate (APP) via spark plasma sintering (SPS)—a 5 wt.% APP content has been identified as optimal for compressive strength (432 MPa, +222% relative to the unreinforced matrix), whereas higher concentrations increase brittleness [18]. These results show that microstructural engineering, control of reinforcement content, and selection of the processing technique are critical variables for optimizing toughness and energy absorption in Al-based MMCs. The elastic energy density per unit volume stored (or elastic energy density) in a material (*Ee*), mainly within the elastic stress fields generated by dislocations during plastic deformation, is given by:

(1) $$E_{e}=\;A\cdot G\cdot b^{2}\cdot \rho \cdot ln(Re/r_{0})$$

In this expression, *G* is the shear modulus (26 GPa for Al), *b* is the magnitude of the Burgers vector (which quantifies the atomic displacement associated with the dislocation and is 0.286 nm for aluminum), and *ρ* is the dislocation density, defined as the total dislocation line length per unit volume of the crystal. The terms *Re* (external cut-off radius) and *r_o_* (internal or core cut-off radius) define the limits of the elastic field of the dislocation, where *r_o_* is usually approximated as *b*, while *Re* can be associated with a characteristic distance such as the grain diameter in nanocrystalline materials or the average dislocation spacing [19]. The constant *A* varies depending on the type of dislocation: *A* = 1/(4π) for a screw dislocation and *A* = 1/(4π(1 − ν)) for an edge dislocation [20]. This stored energy constitutes a fundamental parameter in dislocation physics and microstructural restoration processes, as its release acts as the driving force for phenomena such as recovery and recrystallization [21,22]. The practical applications of elastic energy stored in the stress fields generated by dislocations span various areas of materials science. First, the quantification and characterization of deformed microstructures are achieved through the dislocation density (*ρ*), which allows the estimation of stored energy and the evaluation of microstructural evolution; in this context, X-ray diffraction methods such as complete line profile analysis (CMWP) are employed [23]. Likewise, in modeling strain hardening, the stored energy explains the increase in strength in metals and plays a central role in models such as that of Kocks–Mecking [24]. Similarly, in the design of nanocrystalline materials, the balance between dislocation density and stored energy decisively influences their mechanical properties [25]. The strain energy density (or energy per unit volume) that a material can store up to the yield point (also known as resilience) corresponds to the area under the stress–strain curve in the linear elastic region [26]. For proportional uniaxial loading within the proportional limit (just below the yield point), it can be expressed as:

(2) $$E_{s}=\frac{1}{2}\cdot \sigma_{p}\cdot \varepsilon_{p}$$

In this expression, σ_p_ y ε_p_ represent the stress and the engineering strain within the proportional region of the stress–strain curve. The *Es* (J·m^−3^) quantity quantifies the material’s ability to absorb and release energy without permanent damage; it is useful for comparing materials in scenarios involving impact, vibration, or transient loads.

The mechanical performance of nanocrystalline materials is strongly conditioned by the nature of their interfaces. In particular, the contact zones between metallic and ceramic phases concentrate microstructural heterogeneities that generate local deformations, which in turn have a direct impact on the overall response of the material [27].

To characterize this behavior, the Convolutional Multiple Whole Profile (CMWP) method has been established as a reliable approach, allowing the determination of key microstructural parameters such as dislocation density, dislocation character (edge, screw, or mixed), and the crystallite size distribution [23]. This procedure is based on a mathematical fitting of the full diffraction profile, using models that relate peak broadening to the presence of dislocations and grain size dispersion. Among the derived parameters, the factor *q* is fundamental for identifying the nature of dislocations by comparing experimental values with those theoretically calculated through contrast factors [28]. The theoretical value of parameter *q* is later compared with the experimental value obtained using the CMWP program. When the experimental *q* closely matches the value estimated for edge dislocations, these are classified accordingly. Conversely, if the experimental *q* approaches the characteristic value for screw dislocations, it is assigned that character, as described by T. Ungár and collaborators. Finally, if the experimental *q* value lies within an intermediate range between the edge and screw limits, the dislocations are considered to be of mixed character. Furthermore, the spatial arrangement of dislocations can be evaluated through the dimensionless parameter:

(3) $$M=Re\sqrt{\rho }$$

which describes the degree of correlation among them: values of *M* > 1 indicate dislocations distributed in a random manner, whereas values of *M* < 1 reflect the formation of correlated configurations with short-range strain fields [29]. The effective external cut-off radius of dislocations (*R_e_*) is a fundamental parameter in modeling diffraction profiles broadened by dislocations. In the Krivoglaz–Wilkens theory, *Re* is interpreted as the maximum distance over which the stress field associated with a dislocation retains physical significance before being compensated by other dislocations within the crystal [30]. This radius does not represent a real geometric limit but rather an effective value that integrates the collective effect of long-range dislocation interactions. In practice, *Re* is determined by fitting X-ray diffraction profiles using CMWP. Its magnitude is directly related to the elastic energy stored in the lattice and to the material’s capacity to accumulate internal strains [23]. High *Re* values suggest that dislocation stress fields extend over longer distances, reflecting a less correlated arrangement of defects; in contrast, low values indicate that the fields are rapidly screened due to more ordered configurations with strong spatial correlation. The parameter *q* corresponds to the dislocation character (edge or screw). The theoretical *q* value for dislocations is taken from [29] and compared with the experimental *q* value obtained from XRD analyses. According to the methodology described by T. Ungár et al., the reference values are *q* = 1.31 for pure screw dislocations and *q* = 0.33 for pure edge. In this work the microstructural analysis focused on quantifying the parameters governing the elastic energy stored in the aluminum matrix. Using XRD–CMWP analysis, key dislocation-related parameters (*ρ*, *q*, and *Re*) were determined. Furthermore, the strain field distribution at the Al–Al_4_C_3_ nanorod interface, obtained from HRTEM-based strain mapping, was correlated with these microstructural parameters, thereby establishing a direct link between local interfacial deformation and the specific dislocation arrangements that control the material’s elastic energy storage capacity.

## 2. Materials and Methods

The reinforcement powder of Al_4_C_3_ was obtained through a reaction between Al and C, following the methodology described in a previous study [30]. The reinforcing powder (R) is composed of approximately 51 wt.% Al_4_C_3_ particles with an average size of 13 nm, about 3 wt.% of C phase with an average size of 20 nm, and the remainder consisting of Al. The Al-based composites were produced by mixing Al powder (99.5% purity, Sigma-Aldrich) with 1 and 2 wt.% of R. Each Al–R mixture was subjected to mechanical milling in a high-energy Simoloyer mill for 8 h, using argon as the milling atmosphere and approximately 4 mL of methanol as a process control agent. The device and milling media were made of hardened steel, establishing a ball-to-powder weight ratio of 50:1. Consolidated samples with a diameter of 6 mm and a length of 12 mm were obtained by uniaxial pressing of the powder mixtures for 2 min at approximately 1250 MPa. Subsequently, the samples were sintered at 550 °C for different time intervals: 2 h, 4 h, and 6 h. Table 1 describes the nomenclature, composition, and sintering times of the composites; the first number corresponds to the wt.% of the R powder mixture and the second to the sintering time. The composites were characterized by X-ray diffraction (XRD) (Anton Paar, Boynton Beach, FL, USA) and transmission electron microscopy (TEM) (JEOL Ltd., Tokyo, Japan). The diffraction profiles were recorded using a Philips X’pert powder diffractometer (Anton Paar, Boynton Beach, FL, USA) with a Cu cathode (λ = 0.15406 nm), a step size of 0.02°, and a counting time of 5 s per step. The diffraction profile analysis was applied to determine the crystallite size distribution and dislocation density of the nanocomposites using the CMWP fitting procedure. TEM characterization was performed using a JEOL JEM-2200FS transmission electron microscope (JEOL Ltd., Tokyo, Japan), equipped with a 200 kV field emission gun (FEG) and an energy-dispersive spectrometer (EDS). TEM samples were prepared using the focused ion beam (FIB) technique (JEM-9320FIB, JEOL Ltd., Tokyo, Japan). Compression tests were carried out using a 5-ton universal testing machine at room temperature and a strain rate of 1 × 10^−3^ s^−1^ on cylindrical specimens of 6 mm diameter and 12 mm height, in accordance with ASTM E9-09 [31].

## 3. Results

### 3.1. Elastic Energy Density Analysis

Table 2 presents the values obtained for the samples containing 1, 2, and 3 wt.% of R, derived from the X-ray diffraction patterns using the CMWP technique: the average dislocation density (*ρ*), the experimental parameter (*q*), the average dislocation character (*M*), and the effective outer cut-off radius of dislocations (*R_e_*). The table also includes the results of the elastic energy density (*Es*), obtained using Equation (1).

**Figure 1:**
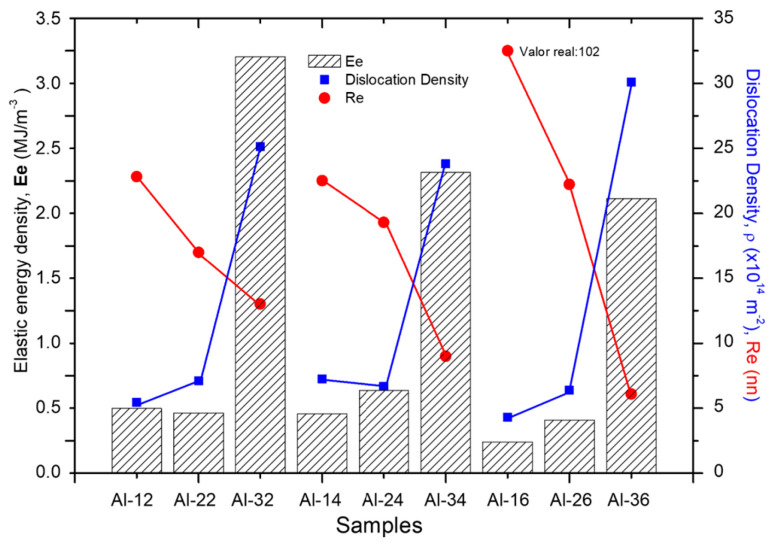
*Ee* vs. *Re* parameter and dislocation density, as a function of composition and sintering time.

Figure 1 presents the calculated *Ee*, derived from XRD data, alongside the dislocation density (*ρ*) and effective outer cut-off radius (*Re*), illustrating their variation with composite composition and sintering time.

### 3.2. Strain Energy Density Analysis

**Figure 2:**
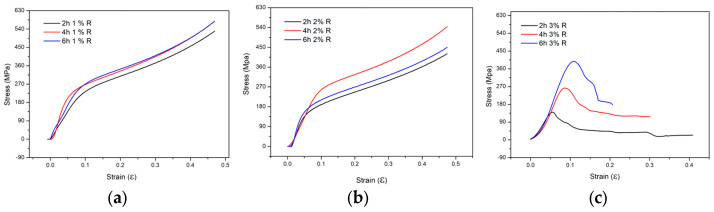
Compression test results of the samples with (**a**) 1 wt.% R, (**b**) 2 wt.% R, and (**c**) 3 wt.% R at 2, 4, and 6 h of sintering.

The graphs in Figure 2 show the behavior of the specimens under compression conditions at different reinforcement contents and sintering temperatures.

Table 3 shows the proportional stress (σ_p_), proportional strain (ε_p_) and strain energy density (*Es*) obtained from the compression tests.

**Figure 3:**
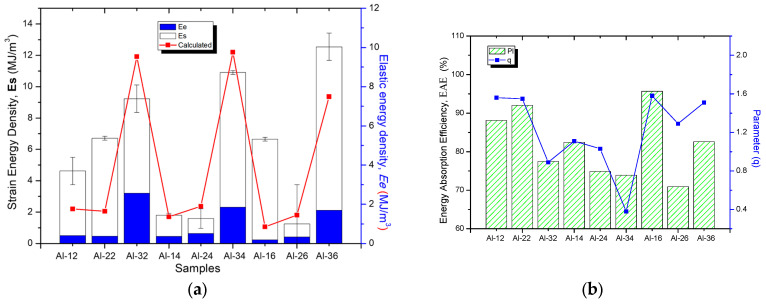
(**a**) Strain energy density, *Es* and Absorbed Energy *Ee*, as a function of composition and sintering time. (**b**) Energy Absorption Efficiency (EAE) as a function of composition and sintering time.

Figure 3a shows the strain energy density (*Es*) calculated using Equation (2) and the compression test data as a function of composition and for different sintering times. For comparison purposes, the graph also includes the elastic energy density (*Ee*) values prior to the compression test, obtained from the XRD analyses. Graphic of Figure 3b shows the energy absorption efficiency (*EAE*) as a function of composition and sintering time. Where *EAE* is expressed as:

(4) $$EAE(\%)=\frac{E_{s}}{E_{e}+E_{s}}\;\times \;100$$

The graph also displays the *q* values, and it can be seen that, for most samples, the *EAE* exhibits a clear correspondence with the *q* values. As a first approximation, a mathematical model is proposed that considers only the proportional increment of *Ee* to estimate the value of *Es*. The calculated stored energy density (*Ecs*) is expressed as:

*Ecs = αEe* (5)

where α = 3.2159. Figure 3a also incorporates the curve corresponding to *Ecs*. The graphs show that *Ecs* curve does not exhibit a clear correlation with *Es* at several points; that is, *Es* does not increase proportionally with the initial *Ee* values. Notable discrepancies are observed in certain intervals; for example, at the points corresponding to samples Al-12, Al-22, Al-32, and Al-16, the model fails to adequately fit the experimental data.

**Figure 4:**
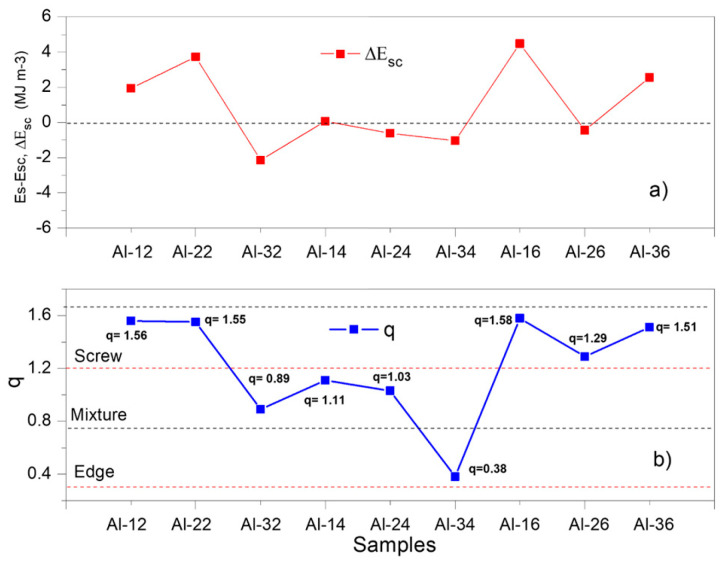
(**a**) Δ*Esc* and (**b**) *q* parameter curves as a function of composition and sintering time.

Figure 4a shows the points corresponding to the difference Δ*Esc* = (*Es* − *Esc*) and compares them with the behavior of the *q* parameter values for each sample, under each processing condition.

### 3.3. Mathematical Model for Estimating Es

**Figure 5:**
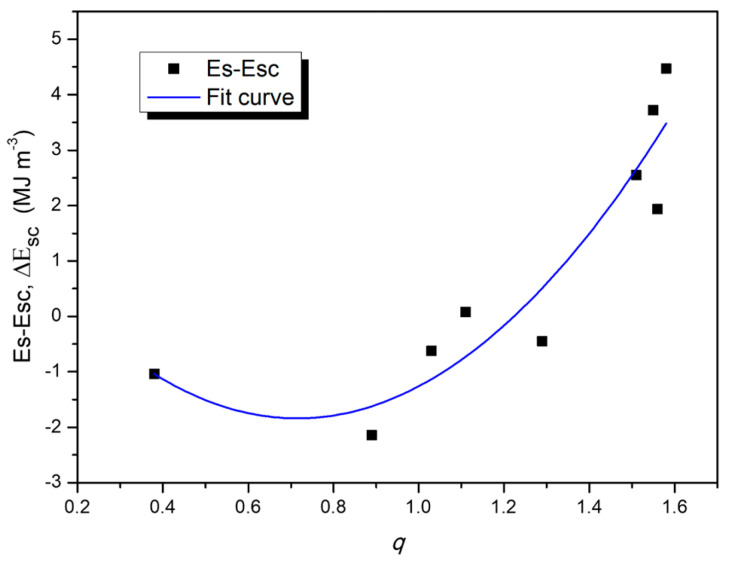
Δ*Esc* curve and fit curve as a function of *q* parameter.

Figure 5 shows the comparison between the experimental Δ*Esc* curve and the fitted curve as a function of the parameter *q*.

**Figure 6:**
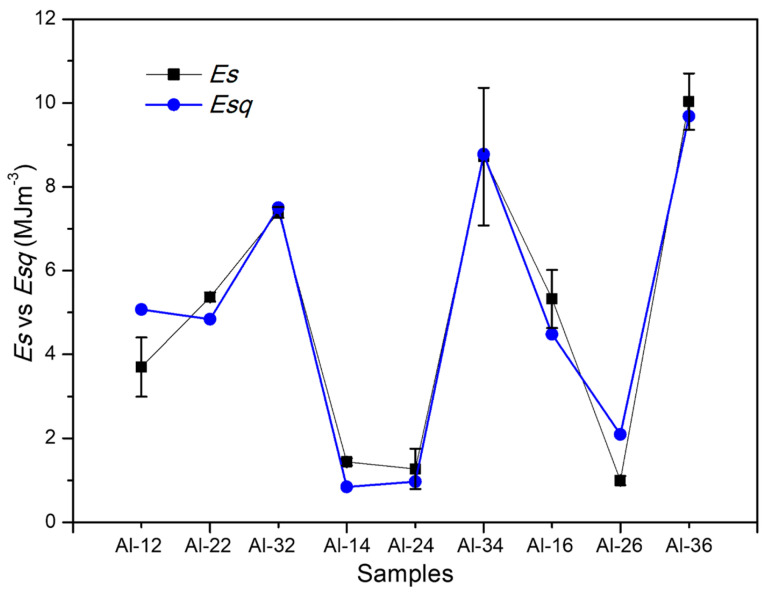
*Es* and *Esq* curves as a function of composition and sintering time.

This behavior suggests a parabolic-type model that explicitly incorporates the initial condition through the stored energy (*Ee*) and also accounts for the effect of the character of the pre-existing dislocations (parameterized by (q)) as a determining factor in the energy absorption capacity. The proposed model is:

(6) $$Esq=\alpha Ee+\beta_{1}+\beta_{2}q+\beta_{3}q^{2}$$

where α = 3.2159, β_1_ = 3.2906, β_2_ = –11.784, and β_3_ = 7.6255. Figure 6 compares the *Es* curve with the *Esq* curve calculated using Equation (6). The graph shows a close correlation between both (R^2^ ≈ 0.93). This result supports the feasibility of predicting the strain energy density (*Es*) in compression tests based on the initial stored energy, estimated through microstructural analysis by XRD.

**Figure 7:**
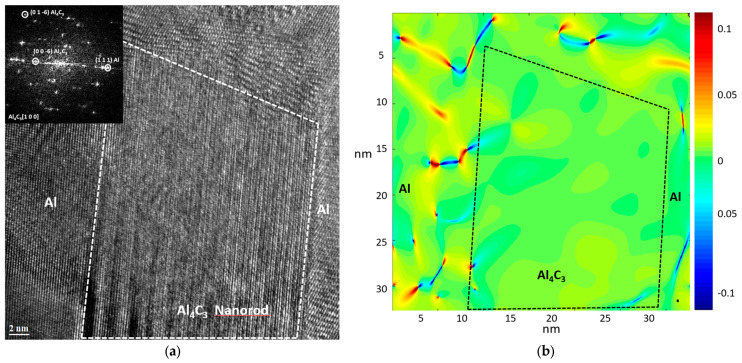
(**a**) High-resolution transmission electron microscopy (HRTEM) image and corresponding digital diffraction pattern of the Al-34 sample, revealing the Al matrix microstructure and a precipitated Al_4_C_3_ nanorod; (**b**) Strain field mapping across the Al matrix and the Al_4_C_3_ nanorod area.

**Figure 8:**
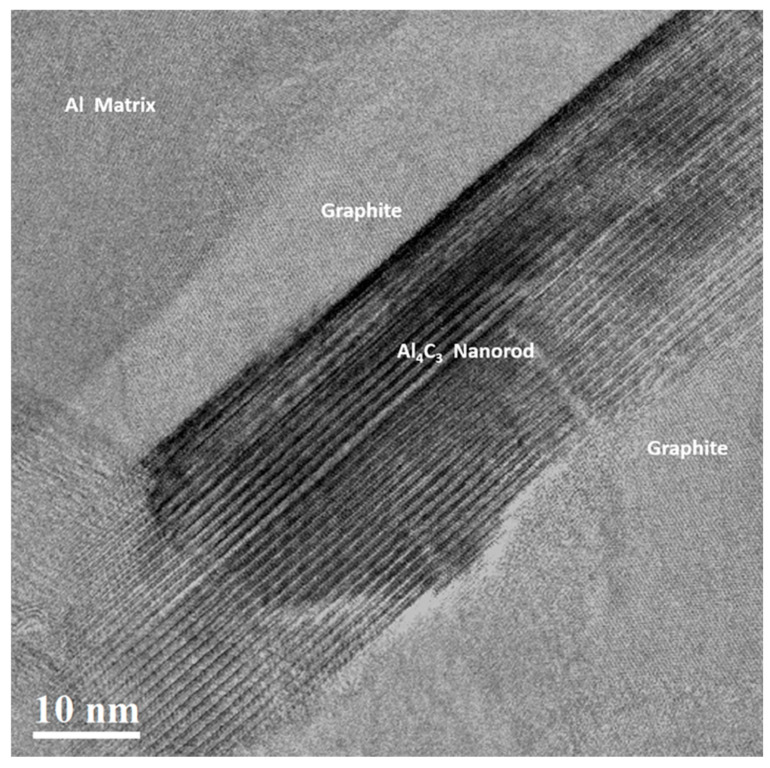
High-Resolution Transmission Electron Microscopy (HRTEM) image of the Al-36 sample.

**Figure 9:**
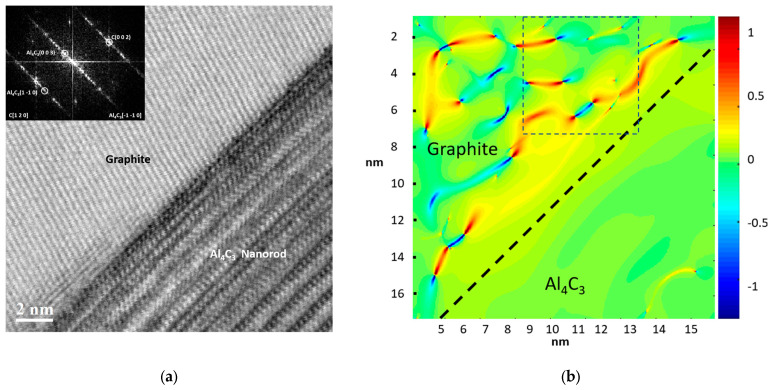
(**a**) High-resolution transmission electron microscopy (HRTEM) image of the Al-36 sample showing the graphite/Al_4_C_3_ nanorod interface, with the corresponding digital diffraction pattern in the inset; (**b**) corresponding strain-field distribution in the surrounding graphite matrix.

A high-resolution transmission electron microscopy (HRTEM) image corresponding to the interface of an Al_4_C_3_ nanorod from the Al-34 sample is shown in Figure 7a. The inserted digital diffraction pattern, obtained through the fast Fourier transform (FFT) of region A, reveals the presence of two distinct zones: one corresponding to the Al phase, oriented along the [0 1 1] zone axis, and another associated with the Al_4_C_3_ phase, oriented along the [1 0 0] zone axis. The image also shows the region corresponding to the Al_4_C_3_ and Al phases, outlined by a dashed line. For the geometric phase analysis (GPA), two primary reciprocal lattice vectors of the Al_4_C_3_ phase, g_1_ = 0 0 6 and g_2_ = 0 1 6, were selected based on the digital diffraction pattern. The distribution of the strain field (ε_xy_), influenced by the presence of dislocations at the interface, is shown in Figure 7b, highlighting the local strain variations induced by the lattice mismatch between both phases. Figure 8 shows an HRTEM image of the Al-36 sample, in which an Al_4_C_3_ nanorod can be observed. The image reveals the presence of a graphite phase located on both sides of the nanofiber, while the Al phase is observed in a more distant region. In the HRTEM image in Figure 9a, a region near the Al_4_C_3_ nanorod can be observed. The inserted digital diffraction pattern, obtained from the FFT of region A, reveals two distinct zones: one corresponding to the Al_4_C_3_ phase, oriented along the [1 1 0] zone axis, and the other corresponding to the graphite phase, oriented along the [1 2 0] zone axis. For the GPA, two primary reciprocal lattice vectors of the Al_4_C_3_ phase were selected, g_1_ = 0 0 3 and g_2_ = 1 1 0, determined from the digital diffraction pattern. The strain field distribution (ε_xy_), due to the presence of dislocations at the interface, is presented in Figure 9b. The image reveals localized strain gradients within the graphite phase induced by its interaction with the stiffer Al_4_C_3_ phase, leading to the formation of dislocation dipoles. These dipoles serve as an efficient elastic accommodation mechanism for the graphite lattice in response to the structural and elastic mismatch imposed by the Al_4_C_3_ nanorod.

**Figure 10:**
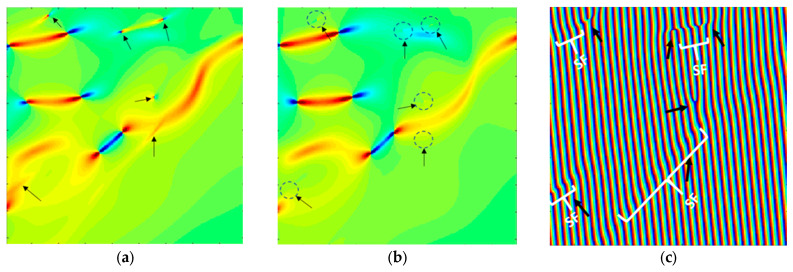
(**a**) Amplified (εxy) strain field distribution from selected area Figure 9b, (**b**) corresponding (εyy) strain field distribution and (**c**) raw phase showing intrinsic stacking faults.

A further insight into the local deformation mechanisms at the graphite/Al_4_C_3_ interface, the strain fields obtained from the geometric phase analysis are examined in detail. Figure 10a shows an amplified view of the (εxy) strain field extracted from the region highlighted in Figure 9, together with the corresponding (εyy) strain distribution (see Figure 10b) and in Figure 10c. Arrows highlight localized strain concentrations that are barely perceptible in the (εyy) map of Figure 10b, while dashed circles indicate regions where localized strain is absent. The raw phase image, in which the presence of intrinsic stacking faults is clearly revealed, correlates with the regions highlighted in Figure 10a,b.

**Figure 11:**
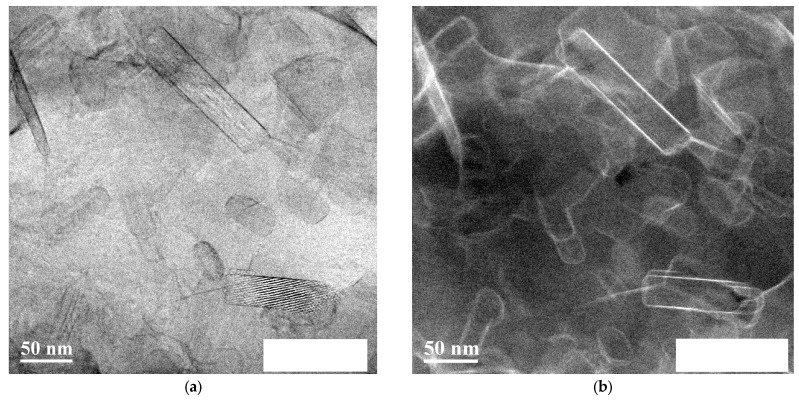
TEM image of the Al-32 sample showing (**a**) the distribution of Al_4_C_3_ nanorods and (**b**) Z-contrast, which reveals the presence of graphite surrounding the Al_4_C_3_ nanorods.

TEM with Z-contrast imaging was employed to further characterize the composite microstructure, including the spatial distribution of the reinforcing phase and the graphite. Figure 11 presents a representative micrograph of the Al-32 sample, highlighting the arrangement of the Al_4_C_3_ nanorods (Figure 11a) and the graphite phase surrounding them, as revealed by the Z-contrast technique (Figure 11b). In the High-angle annular dark-field scanning transmission electron microscopy (HAADF–STEM) image, the bright halo surrounding the Al_4_C_3_ nanorod is consistent with the presence of graphite. As reported for graphitized carbon black, carbon layers locally oriented parallel to the electron beam increase the effective mass–thickness and therefore appear brighter [33].

## 4. Results Discussion

The elastic energy density *Ee* is mainly attributed to the microstructural parameters *ρ*, *G*, *q*, *Re*, and *b* contained in Equation (1), of which *ρ*, *q*, and *Re* were experimentally determined by XRD. In the case of the analyzed samples, it was observed that the dislocation density exerts a significant effect on the value of *Ee*. The high dislocation density observed is attributed to the mechanical milling process, in which numerous linear defects are generated as a consequence of the dispersion of the reinforcement within the aluminum matrix. Additionally, the mismatch in physical properties between the matrix and the reinforcement, particularly in the coefficients of thermal expansion and elastic modulus, generates residual stresses during the sintering and cooling stages. These stresses are mainly relaxed through the nucleation and multiplication of dislocations at the matrix/reinforcement interface. As a result, a higher reinforcement content increases the number of active interfaces and, consequently, the dislocation density in the material. 

Figure 1 shows that larger fractions of reinforcement are associated with higher *ρ* values and, consequently, with increases in *Ee*, in accordance with Equation (1). For example, samples with 3 wt.% reach values between ~2 and 3 MJ/m^3^, which are approximately one order of magnitude higher than those corresponding to compositions with 1 wt.% and 2 wt.% (~ 0.24 and 0.55 MJ/m^3^, respectively). The lowest *Ee* value was verified for the Al-16 sample, sintered for 6 h. This result is consistent, since the sample exhibits a very low dislocation density (~1.8 × 10^14^ m^−2^), and although the *Re* value is quite high (~102 nm), its contribution is marginal due to the logarithmic dependence of *Ee* on *Re* (Note: in the graph, *Re* is plotted as 30 nm for visual clarity). Conversely, the maximum *Ee* value corresponds to the Al-34 sample, resulting from its high dislocation density (~21.3 × 10^14^ m^−2^). Although *Re* is low (~6.5 nm), its effect on *Ee* is negligible compared to the dominant contribution of *ρ*. In fact, a clear inverse relationship is observed between *Re* and the dislocation density (*ρ*), where low *Re* values are systematically associated with high *ρ* values (see Figure 1). This correlation is particularly notable in the Al-32, Al-34, and Al-36 samples. It is important to note that the obtained values (e.g., *Re* = 102 nm for Al-16 and *Re* = 6.5 nm for Al-34) are consistent with their respective crystallite sizes, since the crystallite size represents the upper limit for the expected *Re* values (see Table 2). Furthermore, a progressive reduction in the *Re* parameter is observed in all samples as the reinforcement fraction increases, for each of the sintering times evaluated (2, 4, and 6 h). 

Another factor influencing *Ee* is the parameter *q*, which quantifies the character of the dislocations present in the microstructure (edge/screw ratio). The results of the *q* parameter are presented in Table 2. The table shows that samples with *q* values between 1.29 and 1.58 are associated with a predominantly screw character, while the only sample exhibiting a pure edge character (*q* = 0.33) is the Al-34 sample. The remaining samples, with *q* values ranging from 0.89 to 1.11, are classified as mixed character. The character of the dislocations is incorporated through the factor *A* in Equation (1) and is presented for each *q* value in Table 2. When the dislocation configuration is dominated by screw-character segments, the factor *A* takes a value of approximately *A* ≈ 0.079, which leads to a more pronounced reduction in *Ee* compared with microstructures in which edge dislocations predominate, for which *A* ≈ 0.118. Under configurations where edge and screw dislocations coexist (*A* ≈ 0.099), the value of *Ee* falls within an intermediate range between the cases dominated by either pure character. 

The dislocation density (*ρ*) is a fundamental parameter that quantifies the degree of distortion in the crystal lattice. An increase in *ρ* leads to a proportional rise in the total dislocation line length per unit volume, which directly translates into an increase in the elastic energy density (*Ee*) in the material. On the other hand, the effective range (*Re*) of the stress fields associated with dislocations determines the scale of long-range lattice distortion. A reduction in *Re* spatially limits the influence of these elastic fields, resulting in a decrease in *Ee*. However, when both the dislocation density and the dislocation character (*q*) are approximately similar, the effect of *Re* on *Ee* becomes practically negligible. This is evidenced by the Al-12 and Al-22 samples, where *Re* = 20.02 nm and *Re* = 14.5 nm, respectively, yet the corresponding *Ee* values remain very close (0.311 and 0.464 MJ m^−3^).

Additionally, *q* parameter quantifies the influence of the dislocation character on the elastic energy storage capacity of the material. For instance, edge dislocations exhibit a higher ability to store elastic energy than screw dislocations, as their stress fields are more intense and localized [34]. This greater intensity promotes stronger interactions with microstructural obstacles (solute atoms, precipitates, grain boundaries), which increases local distortion and contributes to hardening and energy storage. In contrast, screw dislocations generate a more distributed pure shear field and can rearrange via cross-slip, so their contribution to energy storage is lower [35]. Overall, microstructures with a higher fraction of edge dislocations tend to exhibit greater accumulated elastic energy, whereas a predominance of screw dislocations reduces it [36].

The graphs in Figure 2 show the behavior of the specimens under compression. In the samples with 1 wt.% and 2 wt.% Al_4_C_3_, a progressive increase in stress is observed as the load on the specimen increases, without a clearly defined fracture point. This indicates a greater capacity for plastic deformation prior to failure. In contrast, the samples with 3 wt.% reinforcement exhibit a different behavior: the stress–strain curves display a well-defined fracture point, evidencing a sudden loss of load-bearing capacity. This result suggests that a higher reinforcement content promotes microstructural mechanisms that limit the ductility of the material. 

Table 3 presents the values of strain energy density, *Es*, calculated using Equation (2) and employing the proportional yield stress (σ_p_) and proportional strain (ε_p_) from the stress–strain curve. Comparable *Es* values are observed between samples with low and high reinforcement because *Es* is a direct function of stress σ_p_ and strain ε_p_, according to Equation (2). In other words, although σ_p_ values are relatively high in the samples with 3 wt.% reinforcement, the corresponding *Es* values are reduced due to the marked decrease in strain, as shown in Table 3. Figure 3 summarizes these results and, for comparison, includes the *Ee* values. At this stage, the deformation is essentially elastic; compression displaces the atoms from their equilibrium positions, storing potential energy that allows full recovery upon load removal. According to the results shown in Figure 3a, the energy *Es* is influenced by the initial elastic energy conditions (*Ee*). That is, samples with a high reinforcement content (3 wt.% R) and a high *Ee* value (average of 2.5 MJ·m^−3^) also exhibit high *Es* values (average of 8.7 MJ·m^−3^). The results show that, although the energy stored during compression, *Es*, is related to the initial *Ee*, its increase is not proportional to the initial *Ee* values. For example, although samples Al-22 and Al-12 showed similar *Ee* values (~0.48 MJ·m^−3^), sample Al-22 reached a significantly higher Es, with 5.36 MJ·m^−3^ versus 3.7 MJ·m^−3^ for sample Al-12. Another case is sample Al-16, which, despite having a very low *Ee* value (0.24 MJ·m^−3^), achieved a considerably high *Es*, close to 5.32 MJ·m^−3^. 

To facilitate understanding of this behavior, the energy absorption efficiency (*EAE*) attained in the elastic region during the compression test was calculated for each sample. Figure 3b shows the *EAE* values for each sample under different processing conditions and also compares them with the *q* parameter values. The graph shows that the samples with the highest *EAE* correspond to Al-22 and Al-16, with approximate values of 92.0% and 95.7%, respectively. In contrast, samples Al-32, Al-24, Al-34, and Al-26 exhibit the lowest *EAE* values, around 70–77%. Therefore, directly applying a proportional model between *Es* and *Ee* (Equation (5)) does not adequately reproduce the experimentally observed behavior. 

The behavior of the calculated curve (*Esc*) according to Equation (5) is shown in Figure 3a. This non-proportionality indicates the suitability of examining the microstructural factors that govern energy storage in the elastic regime for each sample in particular (e.g., dislocation character and density *q*, *ρ* and the effective outer cut-off radius *Re*, etc.). Figure 4a presents the difference curve between the experimentally measured stored energy (Es) and the calculated value (*Esc*) from Equation (5), defined as Δ*Esc* = (*Es* − *Esc*) for each sample. In Figure 4b, *q* ranges are included to characterize the microstructural state according to the predominant dislocation type: mainly screw dislocations (q ≈ 1.3), mainly edge dislocations (q ≈ 0.33), or a balanced mixture of both (q ≈ 0.8). Comparing both plots, it is noted that samples Al-32, Al-14, Al-24, Al-34 and Al-26 exhibit the lowest absolute Δ*Esc* values, between 0.27 and 0.48 MJ·m^−3^. These same samples also exhibit low *q* values close to 1 (0.8–1.3), as shown in Figure 4b, which correspond to the aforementioned samples. Such *q* values are associated with a microstructural state dominated by dislocations of mixed character. Conversely, samples Al-12, Al-22, Al-16, and Al-36 exhibit a substantially elevated Δ*Es*, with values between ~1.9 and 4.45 MJ·m^−3^, together with markedly higher *q* values in the 1.51–1.56 range. This *q* range is associated with a microstructural condition characterized by a greater presence of screw dislocations. The plot also reveals a high correlation between the Δ*Esc* and *q* curves, suggesting that the observed difference may be governed by *q* parameter. 

Figure 5 shows the ΔEs curve and its fit as a function of *q*, where the data exhibit a clear parabolic trend. Figure 6 shows the *Es* curve and its comparison with the stored energy as a function of *q* (*Esq*), calculated using Equation (6). The plot shows a close correlation between *Es* and *Esq*, with R^2^ ≈ 0.95. According to the results obtained, the parameter *q* in Equation (1) (which quantifies the influence of dislocation character) is a determining factor for predicting the behavior observed in the composites. Although this parameter reflects the different energy-storage capacities between edge dislocations, which exhibit more intense and localized stress fields (greater energy-storage capacity), and screw dislocations, whose pure shear stress fields are more diffuse and contribute less to energy storage, the results show that, in this particular system, other microstructural mechanisms related to *q* predominate, in accordance with the proposed model (Equation (6)). The response may be related to the presence of dislocation dipoles and stacking faults. In face-centered cubic (FCC) metals, dislocation motion (particularly that of screw dislocations undergoing cross-slip) produces jogs and generates dipoles formed by edge dislocations of opposite sign. These dipoles act as short-range elastic obstacles that reduce dislocation mobility and contribute to strain hardening [37]. 

A decrease in *Re* with increasing reinforcement indicates a stronger tendency for dislocation dipole formation. Low *Re* values imply rapid cancellation of strain fields, which limits dislocation motion and multiplication; consequently, the density of mobile dislocations decreases, resulting in reduced stored elastic energy [38]. This stored energy plays a critical role in redistributing and dissipating stresses during deformation; therefore, its reduction leads to strain localization and shortens the plastic stage prior to fracture. As a result, the total energy absorbed before failure decreases, leading to reduced material toughness. This behavior is observed in the 3 wt. % samples subjected to compression (see Figure 2c). In particular, the microstructural characterization of the Al-34 alloy shows a predominance of edge dislocations (see Table 2). Since dipoles are mainly formed by pure edge dislocations [39], it is reasonable to expect that this sample contains a high density of dislocation dipoles. This condition limits the ability of the material to store elastic energy, which is reflected in the *EAE* value of the Al-34 sample (~74%), the lowest among the specimens reinforced with 3 wt.% (see Figure 3b). 

Figure 7b shows the HRTEM image of the Al-34 sample where the strain field distribution (ε_xy_) is observable, which arises primarily from dislocation dipoles distributed around an Al_4_C_3_ nanorod. On the other hand, the Al-36 sample exhibits an improvement in *EAE* of ~82.6%. According to the XRD analysis, the microstructure of this sample is associated with a higher dislocation density (~2.7 × 10^15^ m^−2^), with a predominantly screw character (see Table 2). According to the theory, higher fraction of screw dislocations reduces the formation of dislocation dipoles, which would otherwise hinder dislocation multiplication during plastic deformation. According to dislocation theory, a higher fraction of screw dislocations is generally associated with a reduced formation of dislocation dipoles. However, the observed decrease in *Re* with increasing reinforcement content indicates a stronger tendency for dipole formation. This sample exhibits the lowest *Re* value (~3.58 nm) and would therefore be expected to show enhanced dipole formation, which in turn hinders dislocation multiplication during plastic deformation. However, this same sample presents the highest dislocation density, suggesting that additional mechanisms are involved in the generation and accumulation of defects. 

A distinctive feature of the Al-36 sample is the presence of graphite at the Al–Al_4_C_3_ interface. HRTEM analysis confirms this, revealing graphite located at the interface of the Al_4_C_3_ nanorods (see Figure 8). The formation of this graphite phase can be attributed to diffusion processes associated with the prolonged sintering times. Therefore, the high dislocation density at the interface can reasonably be attributed primarily to the thermal mismatch between the graphite and the metallic matrix that develops during cooling. The HRTEM image in Figure 9a shows a region near an Al_4_C_3_ nanorod; the phase present at the interface corresponds to graphite, whose observed planes are associated with the basal (002) plane. In turn, Figure 9b shows the distribution of the strain field (ε_xy_), associated with the presence of well-defined dislocation dipoles, as well as dipoles that exhibit reduced strain fields. Figure 10a shows the distribution of strain fields (ε_xy_) in a specific region of image of Figure 9b. In the image, arrows indicate the presence of strain concentrations that are barely perceptible in the (ε_yy_) map shown in Figure 10b. Normal strain components (ε_yy_) were analyzed to emphasize deformation fields typically dominated by edge dislocations, while the shear component (ε_xy_) was used to highlight contributions more sensitive to screw dislocations. Since strain fields are inherently tensorial and mixed dislocations can contribute to all components, the comparison between ε_yy_/ε_xy_ and ε_yy_ provides a practical criterion for identifying dominant deformation trends associated with dislocation character. The presence of these local strain concentrations, which are only faintly discernible in the ε_xy_ map, is consistent with the existence of extended dislocations, possibly associated with the dissociation of screw segments into partial dislocations separated by stacking faults. In the raw phase image (see Figure 10c), regions consistent with stacking faults are identified, and these correlate with the presence of such dislocations. These configurations may correspond to partial dislocations of mixed character (edge + screw), in which the local elastic field combines a dominant rotational shear component with weaker normal contributions. This behavior has been reported both experimentally and through atomistic simulations [38,40]. 

The presence of screw dislocations in systems containing graphite or in the vicinity of interfaces has been previously reported in the literature [41]. The dissociation of these dislocations into mixed-character partials, observed in the graphite phase, may account for both the high dislocation density and the reduction in the system’s energy, through the formation of stacking faults that relax the core field and redistribute the shear strain over a wider glide region [42,43]. This condition accounts for the improved energy storage efficiency and the increased toughness of the material in samples that exhibit a predominantly screw-type dislocation character. Previous studies have documented the addition of elements such as lithium to aluminum alloys with the aim of reducing the stacking fault energy (SFE) and promoting the emission of partial dislocations. This mechanism facilitates the nucleation of stacking faults and twinning planes, which subsequently activate additional plastic deformation modes, thereby enhancing ductility [44]. In Figure 11b, the graphite phase surrounding the Al_4_C_3_ nanorods is clearly revealed by the Z-contrast technique. This microstructural configuration, characterized by well-dispersed Al_4_C_3_ nanorods embedded in a graphite-containing matrix, is expected to play a key role in the mechanical response of the composite, particularly in terms of energy absorption and damage tolerance.

## 5. Conclusions

- Compression tests were conducted to determine the stored strain energy density (*Es*) in the Al/Al_4_C_3_ composites, and these results were compared with the initial elastic energy density (*Ee*) calculated from microstructural parameters obtained by X-ray diffraction (XRD).
- A mathematical model based on dislocation character was developed, allowing us to establish that the difference observed between *Es* and the proportional increase in *Ee* associated with elastic deformation of the Al matrix depends on the distribution of energy stored in microstructural defects generated during the fabrication processes.
- These microstructural defects are directly related to the dislocation character, as reflected by the *q* parameter obtained from CMWP analysis. Samples with a dislocation density dominated by screw segments exhibited a more efficient energy redistribution during processing, attributed to the dissociation of dislocations into partials and the formation of stacking faults, resulting in an increase in the energy absorption efficiency (*EAE*).
- In contrast, samples containing predominantly edge dislocations showed a higher proportion of dislocation dipoles, which generate highly localized strain fields and consequently reduce the *EAE*.
- The increased presence of screw dislocations is associated with the formation of graphite at the Al_4_C_3_ nanorod interfaces, a phase that develops as a consequence of prolonged sintering times.
- The generation of dislocations at both the graphite/Al_4_C_3_ interface and the Al_4_C_3_/Al matrix interface originates mainly from the thermal mismatch among the graphite phase, Al_4_C_3_, and the aluminum matrix during cooling.
- HRTEM analyses revealed the presence of graphite regions in the vicinity of Al_4_C_3_ nanorods, which, together with thermal mismatch during cooling, promote the activation of dislocation mechanisms dominated by screw segments, their extension into partial dislocations, and the formation of stacking faults. These processes facilitate a more efficient redistribution of stored energy and are reflected in an improvement in the composite toughness.
- For samples with 1 wt.% and 3 wt.% reinforcement, a sintering time of 6 h promoted an increase in *EAE*, an effect associated with enhanced graphite formation during prolonged sintering. In contrast, for samples with 2 wt.% reinforcement, the maximum *EAE* was achieved at a sintering time of 4 h, suggesting the existence of an optimal graphite formation condition under these processing parameters.

## Figures and Tables

**Table 1:** Compositions for studied Al–Al_4_C_3_ samples and nomenclature (in wt.%) ^†^.

Nomenclature	Al (wt. %)	R, Mixture Powder (wt. %)	Sintering Time (h)
Al-30	99	3	0
Al-32	99	3	2
Al-34	99	3	4
Al-36	99	3	6

^†^ Composition for 1 a 2 wt. % R was previously published by the same author in [32].

**Table 2:** Dislocation density and microstructural parameters obtained from X-ray diffraction, constant A and elastic energy density (Ee).

Composition	Dislocations *ρ* (10^14^ m^−2^)	<x> area (nm)	*q*	Dislocation Character	*A*	*M*	*Re* (nm)	Elastic Energy Density, *Ee* (MJ m^−3^)
Al-12	4.75 ^†^	172.5 ^†^	1.56 ^†^	Screw	0.079	0.343 ^†^	20.02 ^†^	0.311
Al-14	2.9 ^†^	154.0 ^†^	1.11 ^†^	Mixture	0.099	0.443 ^†^	20.33 ^†^	0.502
A1-16	1.8 ^†^	184.3 ^†^	1.58 ^†^	Screw	0.079	1.37 ^†^	102.38 ^†^	0.240
Al-22	4.6 ^†^	139.2 ^†^	1.55 ^†^	Screw	0.079	0.312 ^†^	14.50 ^†^	0.464
AL24	4.16 ^†^	199.3 ^†^	1.03 ^†^	Mixture	0.099	0.34 ^†^	16.81 ^†^	0.428
AL26	3.87 ^†^	271.8 ^†^	1.29 ^†^	Screw	0.079	0.38 ^†^	19.72 ^†^	0.408
Al-32	22.63	62.3	0.89	Mixture	0.099	0.5	10.52	2.147
Al-34	21.30	43.3	0.38	Edge	0.118	0.29	6.49	3.077
Al-36	27.60	62.9	1.51	Screw	0.079	0.188	3.58	2.115

^†^ Experimental results previously published by the same author in [32].

**Table 3:** Proportional stress (σ_p_, ε_p_) and strain and *Es* along with their corresponding standard deviations (SD).

Composition	Proportional Stress σ_p_ (Mpa)	SD	Proportional Strain, ε_p_	Strain Energy Density, *E_s_* (MJ m^−3^)	SD
**Al-12**	131.45	24	1.116	3.70	0.7
**Al-14**	117.82	1	1.56	1.44	0.1
**A1-16**	177.14	29	1.58	5.32	0.7
**Al-22**	97.99	9	1.55	1.27	0.1
**AL24**	180.33	18	1.03	5.37	0.5
**AL26**	73.06	5	1.29	1.00	0.1
**Al-32**	214.83	80	0.89	7.39	0.1
**Al-34**	297.77	55	0.38	8.72	2
**Al-36**	326.35	14	1.51	10.04	0.7

## Data Availability

The original contributions presented in this study are included in the article. Further inquiries can be directed to the corresponding authors.
